# Anorexia Nervosa and Gender Dysphoria: A Clinical Case

**DOI:** 10.1192/j.eurpsy.2022.1496

**Published:** 2022-09-01

**Authors:** J. Martins, R. Vaz, A. Costa, J. Brás, R. Sousa, J. Abreu, E. Almeida, T. Casanova

**Affiliations:** Centro Hospitalar Tondela-Viseu , Departamento De Psiquiatria E Saúde Mental, Viseu, Portugal

**Keywords:** Gender Dysphoria, eating disorder

## Abstract

**Introduction:**

Eating disorders (ED) and gender dysphoria (GD) are associated with a change in body perception. Therefore, body dissatisfaction plays a common and central role in these disorders. In GD, body image concerns are related to the features of the biological sex. In ED, body dissatisfaction comes from a distorted perception of weight and body shape and plays an important role in the development and maintenance of the psychopathology.

**Objectives:**

To present and discuss the clinical case of a patient with a previous diagnosis of GD who presented with a clinical condition suggesting a restrictive anorexia nervosa (AN).

**Methods:**

Patient´s clinical files consultation and literature review using Pubmed and the keywords: eating disorders and gender dysphoria.

**Results:**

We present the case of a 25-year-old patient who was living in a shelter for victims of domestic violence and was admitted for severe restrictive AN. The patient was discharged after 40 days and medicated with sertraline, diazepam and olanzapine, as well as her previous medication (hormonal therapy): cyproterone, finasteride, estradiol, oxybutynin.

**Conclusions:**

Although studies on this subject are still scarce, there has been some progress and the literature recognizes the coexistence of these conditions. However ED symptoms in patients with GD could have a different meaning: they may represent a dysfunctional coping strategy adopted to block features of the biological sex. Therefore health professionals may take a more holistic approach to body image. Additional studies will be necessary, allowing the establishment of cause-consequence interactions between weight loss and psychopathology related to GD.

**Disclosure:**

No significant relationships.

